# 3D printed guide tube system for acute Neuropixels probe recordings in non-human primates

**DOI:** 10.1088/1741-2552/acd0d7

**Published:** 2023-05-11

**Authors:** Devyn Lee Bauer, Ben Pobiel, Kathryn Hilber, Ajay K Verma, Jing Wang, Jerrold Vitek, Matthew Johnson, Luke Johnson

**Affiliations:** 1 Department of Biomedical Engineering, University of Minnesota, Minneapolis, MN, United States of America; 2 Department of Neurology, University of Minnesota, Minneapolis, MN, United States of America

**Keywords:** Neuropixels, 3D printing, acute recordings, rhesus macaques, CAD, electrophysiology

## Abstract

*Objective.* Neuropixels (NP) probes are a significant advance in electrophysiological recording technology that enable monitoring of hundreds of neurons in the brain simultaneously at different depths. Application of this technology has been predominately in rodents, however widespread use in non-human primates (NHPs) such as rhesus macaques has been limited. In this study we sought to overcome two overarching challenges that impede acute NP implantation in NHPs: (1) traditional microdrive systems that mount to cephalic chambers are commonly used to access cortical areas for microelectrode recordings but are not designed to accommodate NP probes, and (2) NHPs have thick dura mater and tissue growth within the cephalic chambers which poses a challenge for insertion of the extremely fragile NP probe. *Approach.* In this study we present a novel NP guide tube system that can be adapted to commercial microdrive systems and demonstrate an implant method using the NP guide tube system. This system was developed using a combination of CAD design, 3D printing, and small part machining. Software programs, 3D Slicer and SolidWorks were used to target cortical areas, approximate recording depths and locations, and for in-silico implant testing. *Main results.* We performed *in vivo* testing to validate our methodology, successfully implanting, explanting, and reimplanting NP probes. We collected stable neurophysiological recordings in the premotor cortex of a rhesus macaque at rest and during performance of a reaching task. *Significance.* In this study we demonstrate a robust Neuropixels implant system that allows multiple penetrations with the same NP probe and share design files that will facilitate the adoption of this powerful recording technology for NHP studies.

## 1. Introduction

**Figure 1.**
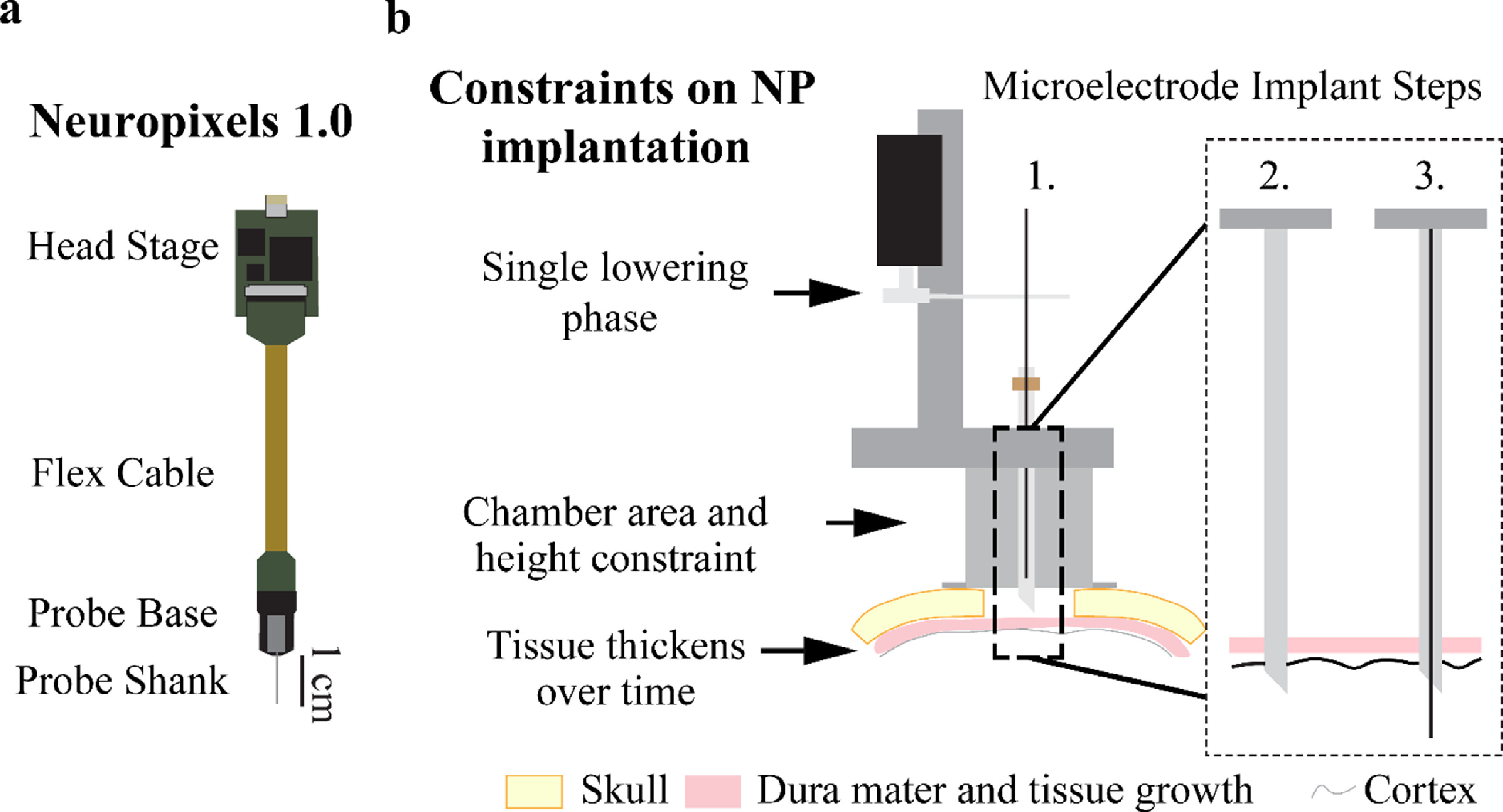
Conventional microelectrode recordings and associated design constraints imposed by utilizing a microdrive approach for Neuropixels probes. (a) Diagram of Neuropixels probe and headstage. (b) Standard microelectrode implantation steps and constraints traditional microelectrode implantation impose on a Neuropixels probe. Microelectrodes use a single lowering phase dedicated to lowering the microelectrode through a pre-placed guide and puncture cannula while a Neuropixels probe requires two lowering phases. The chamber’s 19 mm inner diameter and 25 mm height constrains targetable cortical area since the Neuropixels probe base has a width of 10 mm. Dura mater and periosteum thicken over time after a craniotomy (skull thickness approximated to be 3 mm). Microelectrode implant steps are: 1. Attach the microdrive (modeled after a Narishige microdrive) to the recording chamber, 2. Puncture the dura mater with a guide cannula to provide passage for the microelectrode through the dura mater into the brain, and 3. Descend the microelectrode into the brain to record neuronal activity.

Recently developed Neuropixels (NP) probes (figure 1(a)) are a major advancement in recording technology that enable monitoring the activity of hundreds of neurons simultaneously (Jun *et al* 2017). Manufactured with complementary metal-oxide-semiconductor (CMOS) technology, these probes contain 960 recording sites (with 384 selectable for recording at any given time) along a 10 mm shank. Researchers have implanted these probes both acutely and chronically in head fixed and freely moving mice and rats, demonstrating the NP probe’s utility in characterizing large neuronal populations with high spatiotemporal precision (Jun *et al* 2017, Juavinett *et al* 2019). There are fewer reports of NP use with non-human primates (NHPs; e.g. rhesus macaques), however, and limited information regarding the implantation methodology (Trautmann *et al* 2019, Wang *et al* 2021). A robust NHP NP implant system capable of multiple penetrations with the same NP probe would be of considerable utility to facilitate the adoption of this recording technology in NHP studies.

A common approach used for acute microelectrode recordings in NHPs includes a chronically implanted cranial chamber placed over a craniotomy (figure 1(b), Step 1). Chamber mounted drive systems (Kelly *et al* 2007, Miocinovic *et al* 2007, Johnson *et al* 2015, Deffains *et al* 2016, Prescott *et al* 2017) are affixed to the chamber and used to lower single or multiple microelectrodes into brain tissue. In figure 1(b), a Narishige microdrive (MO-97) is depicted for reference. While metal microelectrodes may be able to penetrate newly exposed dura mater, it is often necessary to pass the microelectrode through a cannula that punctures the dura mater and helps prevent damage to the microelectrode as illustrated in figure 1(b), Steps 2–3.

The first obstacle to implanting a NP in a NHP is that the NP probe has a fragile silicon shank incapable of penetrating the thicker NHP dura mater. Even when implanting the NP probe after a new craniotomy, a dura mater puncture (or cut) must be made to ensure the probe shank does not break (Dutta *et al* 2019, Trautmann *et al* 2019). Furthermore, tissue growth occurs on top of the NHP dura mater after the craniotomy (figure 1(b)) which makes implantation more difficult. Once the dura mater thickens and tissue growth occurs, a guide cannula is required to ensure the probe shank does not break while passing through the dura mater. Therefore, in most implant scenarios, the NP probe needs a guide tube for safe penetrations and probe reuse.

The second obstacle to implanting a NP probe is holding the probe shank in alignment with the guide tube during lowering. In a traditional implantation method, a guide cannula punctures the dura mater and is left in place (figure 1(b), Step 2) for the longer microelectrode to pass through the cannula into the brain (figure 1(b), Step 3). The traditional approach, as described, cannot work seamlessly with a NP probe given a guide tube must be held distal to the probe base with a secondary apparatus (figure 1(a)). Additionally, this apparatus must accommodate the probe base while maintaining the alignment of the probe shank to the guide tube. Secondary to holding the guide tube in alignment with the NP probe shank, the guide tube must be long enough to traverse the dural tissue to ensure probe safety but short enough to maximize the length of the NP probe shank inserted into the brain.

The third obstacle to implanting the NP probe is the size constraint a recording chamber imposes on the NP probe and guide tube holding apparatus (figure 1(a)) Given that the NP probe takes up more space within the chamber (figure 1(b)) than a microelectrode and requires a separate apparatus to hold the guide tube, the size of both systems and how they function together within the cephalic chamber must be accounted for in the design of an effective NP implant system. Increasing the size of the holding apparatus minimizes the targetable area within the cephalic chamber, therefore an optimal NP implant method would maximize the targetable area by minimizing the NP implant footprint within the chamber.

To overcome these challenges and enable repeat acute recordings in NHPs, we detail a method that retrofits an open chamber electrode lowering system to hold and safely implant a NP within a cephalic chamber. The NP guide tube system’s novelty comes from pre-aligning a NP probe to a guide tube that bridges the dura mater and protects the probe, stabilizing local tissue during data collection, and its adaptability to most open chamber, two stage microdrive lowering systems. Using this method of implantation, we simultaneously recorded hundreds of neurons during reaching tasks, targeted multiple areas within a recording chamber, and successfully reimplanted both commercially available NP mouse 1.0 and NHP 1.0 probes. In the following sections, we describe the components that make up the NP guide tube system, the methodology for driving the NP probe into the brain, the approach for precise cortical targeting, proof of concept testing in a rhesus macaque animal model, and present neuronal responses during a reaching task.

## 2. Methods

### 2.1. Animal preparation

All experimental procedures were approved by the Institutional Animal Care and Use Committee and complied with United States Public Health Service policy on the humane care and use of laboratory animals. A single adult female rhesus macaque (*Macaca mulatta*, animal J, 21 years) was used for this study. Surgical placement of recording chambers, screws, and a head stabilization device occurred under aseptic conditions using isoflurane anesthesia. Cephalic recording chamber implant locations were determined using the surgical planning software Cicerone (Miocinovic *et al* 2007) and implanted bilaterally over the dorsal premotor cortices. *In-vivo* testing using the NP probe was performed after this animal completed a separate study. The dura mater at the time of testing, approximately two years and three months after the craniotomy surgery, was thick and no underlying cortical sulci were visible. The primate was trained to perform a food reward reaching task where a successful trial consisted of the NHP reaching from the starting position (a hand resting location in front of the primate) to the food reward, bringing the food to its mouth, and returning its hand to starting position.

### 2.2. Microdrive selection

**Figure 2.**
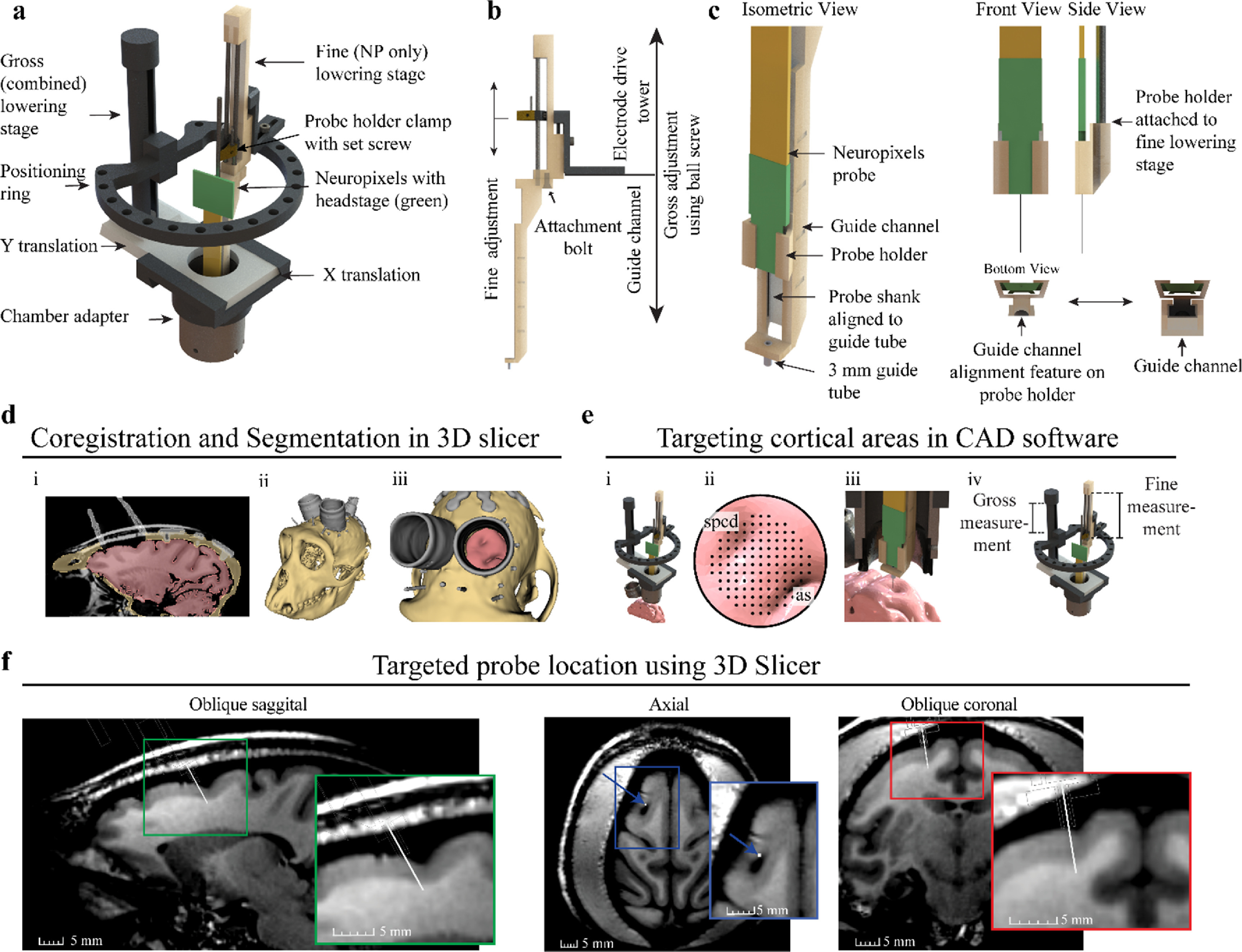
Two-stage Neuropixels implantation system and cortical targeting overview. (a) CAD rendering showing components of the Alpha Omega microdrive system with custom Neuropixels guide tube system. (b) Gross adjustment of the assembly is performed by turning the gross lowering stage ball screw, attached to the ring platform, and fine adjustment is performed moving the fine stage clamp with the electrode positioning system. The fine adjustment independently controls the movement of the Neuropixels probe. (c) Design modifications adapting the Neuropixels to the two-stage microdrive system. The Neuropixels probe is placed into the probe holder and manually aligned to the 3 mm guide tube, which bridges the dura mater and periosteum growth. The probe holder fits into vertical guide channel, maintaining alignment of probe shank to guide tube during fine stage lowering. (d) Primate anatomy is imaged using 7 T MRI, registered to CT, and segmented in 3D slicer showing the: (i) coronal segmentation scans, (ii) whole 3D segmentation, and (iii) view through recording chamber. (e) (i) Microdrive model fixed to chamber in CAD software, including segmentation generated previously. (ii) Targeting location is determined using a grid rendered over the region of interest. The superior precentral dimple (spcd) and arcuate sulcus (as) are labeled. (iii) Both stages are lowered until the probe is inserted into the target. (iv) Gross and fine stage depth estimates are determined through measurements of the model (f). Approximate Neuropixels probe trajectory and recording locations can be visualized by importing relative model positions from CAD software back into 3D slicer, shown here in oblique sagittal, axial, and oblique coronal 7 T MRI views.

Our general approach was to design a ‘Neuropixels guide tube system’ that could interface with an existing microdrive and enable alignment and insertion of a NP probe through a small cannula positioned within the cranial chamber. The Alpha Omega Flex MT microdrive (figure 2(a)) was identified to be well suited for implanting a NP because the Flex MT microdrive has an open chamber adapter, a positioning ring that provides flexibility for positioning the NP probe within the chamber, and it has a lowering stage for the NP probe and one for the guide tube. With respect to moving components, the gross adjustment stage translates the ‘positioning’ ring and attached fine adjustment stage by hand turning a ball screw. Given the measurement scale on the drive tower, the depth of the gross adjustment stage can be measured with 0.5 mm resolution. The fine adjustment stage, also known as the electrode drive tower, translates the NP probe along a guide channel with micron resolution via the attached motorized microdrive, also called the electrode positioning system (EPS). Used in conjunction, both stages provide independent control of the guide tube and NP probe (figure 2(b)). Finally, the electrode drive tower can be placed at multiple positions around the positioning ring to increase the targetable area on the cortical surface. A puncture assist guide from Narishige microdrive was used to assist dura puncturing while maintaining stereotaxic and chamber alignment (further details described below in section: ‘Neuropixels Implantation Sequence—Aligning the Narishige and the Flex MT system’).

### 2.3. 3D printed NP guide tube system

The NP guide tube system was designed in two parts, a probe holder and guide channel. The probe holder and guide channel were designed in SolidWorks and printed using FormLabs 3B stereolithography (SLA) material with a printing tolerance of 50 *µ*m. Designs are provided in supplementary material. The probe holder was designed to fit around the probe base. The probe was manually slid into the probe holder until the probe base was at the distal end of the probe holder. Hot melt adhesive (Surebonder, 725R10-1P) was applied to the probe and probe holder to fix the probe in position. The probe holder attaches to the probe (figure 2(c), front and side view) and acts as an alignment carriage to keep the probe shank centered within the guide channel (figure 2(c), bottom view). The guide channel functions as a path for the probe holder and holds a 4.5 mm long, 18-gauge guide tube. The top 1.5 mm of the guide tube is seated in the guide channel platform and the remaining 3 mm of guide tube was exposed for bridging tissue (figure 2(c), isometric view). The guide tube was fabricated by removing the tip from an 18-gauge stainless steel needle (Monoject 18 × 1A) via a grinding stone on a Dremel rotary tool. Another stone was used to create a bevel on the edge of the remaining tube and smoothed with high grit (fine) sandpaper to remove any residual roughness left over from the beveling process. The guide tube gauge provided probe clearance and matched the puncture tube gauge. The guide tube was glued into a pre-aligned hole in the platform at the bottom of the guide channel. Additionally, the small square platform surrounding the guide tube (figure 2(c), isometric view bottom left) was designed to stabilize tissue surrounding the probe shank. The NP probe, probe holder, and guide channel have a total cross-sectional area of 67 mm^2^.

To integrate the NP guide tube system into the Flex MT microdrive, the guide channel was bolted into the base of the electrode drive tower using an existing hole and bolt (figure 2(b), attachment bolt). The probe holder with the attached probe was then inserted into the guide channel and clamped into the electrode drive tower. Prior to experiments, the probe shank’s alignment to the guide tube was confirmed by visually inspecting the probe shank as it slowly passed through the guide tube, ensuring that the NP shank was centered as it entered and exited the guide tube. Theoretically, given a guide tube inner diameter of 0.838 mm and NP shank length of 10 mm, the maximum allowable angular deflection is 2.1°. If adjustments were required, the hot melt adhesive was rewarmed to reposition the probe shank. After confirming proper alignment, visualization of the probe shank passing through the guide tube during *in-vivo* implantation was not required.

### 2.4. Planning and targeting with the NP guide tube system

To target cortical areas and perform in-silico testing, 3D Slicer (Fedorov *et al* 2012) and 3D CAD software SolidWorks were used due to their complementary functionality. 3D Slicer provided excellent handling of the medical images and SolidWorks provided complete control over the microdrive model. Additionally, both software can import and export stereolithography files allowing for efficient transfer of models between software.

3D Slicer was used to align the primates 7 T magnetic resonance imaging (MRI) and computed tomography (CT) images and segment bone (tan), brain (pink), and instrumentation (grey) regions (figure 2(d), i-iii). Segmentations were spatially fixed together in 3D Slicer and exported into SolidWorks. The 3D model of the Flex MT microdrive was attached to the cephalic chamber segmentation for targeting with NP guide tube system. This approach provided the ability to manipulate all microdrive components (figure 2(e), i) to target various cortical locations (figure 2(e), ii-iii). Utilizing the radial targeting of the ring, the targetable area was 76% of the chamber (19 mm ID) or a 8.79 mm targetable radius from the chamber center (figure 2(e), ii). Since the Flex MT model has a 1:1 (real life) scale, *x*–*y* coordinates for the guide tube and gross stage translation measurements were collected to inform the puncture *x*–*y* coordinates and physical gross lowering stage movements during *in-vivo* implantation (figure 2(e), iv). After planning and targeting within SolidWorks, the microdrive model was exported in the fully implanted position into 3DSlicer and attached to the cephalic chamber to visualize the probe depth across MRI scans (figure 2(f)).

### 2.5. NP implantation sequence

Radial targeting with the NP guide tube system increased the accessible area within the chamber but required the alignment of the NP guide tube system to the puncture site in the dura. A benchtop testing apparatus consisting of a cephalic chamber, paper to mimic the dura mater, and support structure was used to perform alignment of the NP guide tube system to the puncture hole. The paper within the testing apparatus was punctured using the Narishige guide cannula puncture apparatus at the *x*–*y* coordinates determined from the 3D model. The Alpha Omega Flex MT system was then placed on the testing apparatus and the guide tube on the NP guide system was aligned to the punctured hole in the paper. *In-vivo* implantation occurred after the two systems were physically aligned and tested in the benchtop apparatus. Designs for benchtop testing apparatus are included in supplementary material.

**Figure 3.**
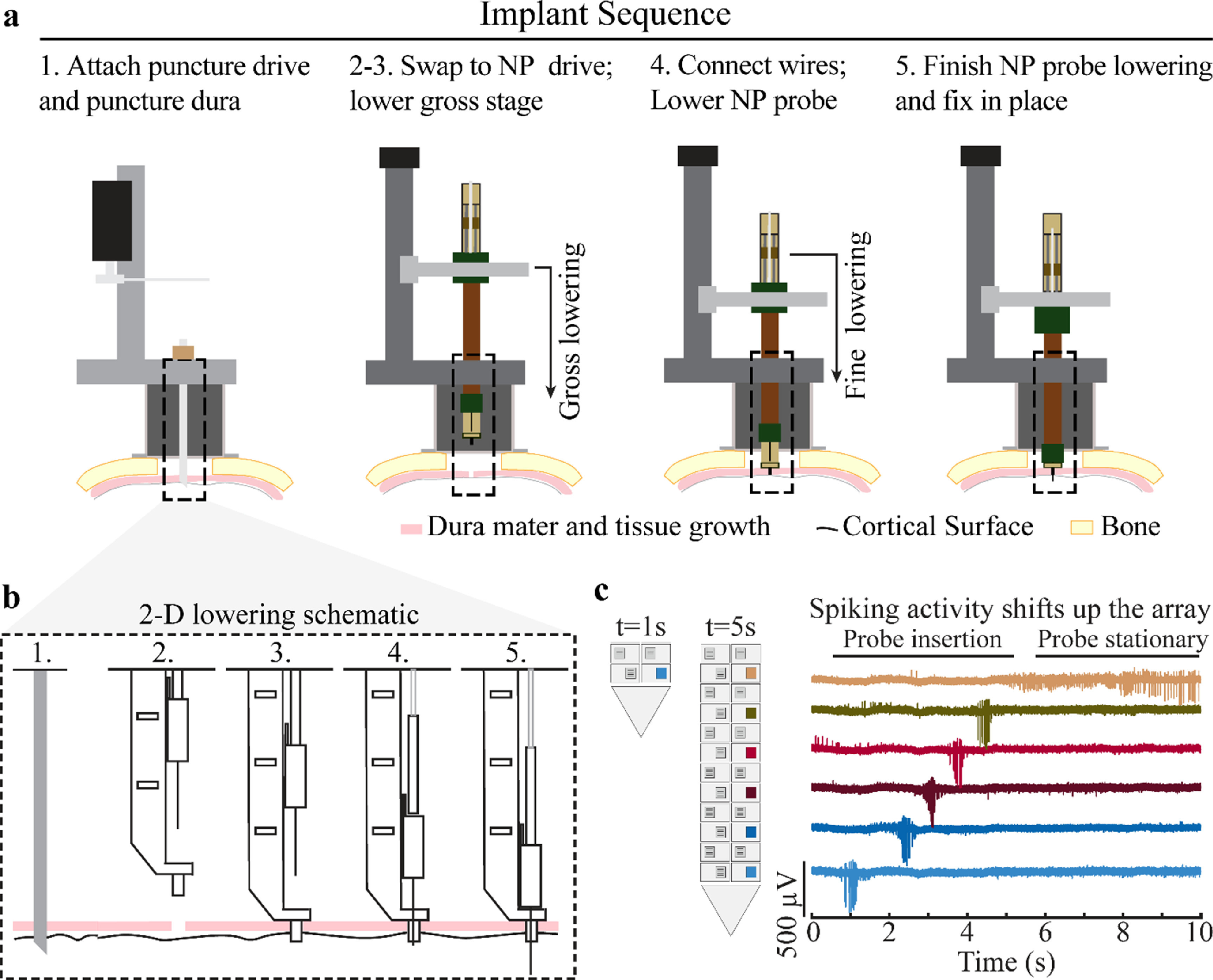
Implantation sequence. (a) Dura mater is punctured using the Narishige system (step 1). The Flex MT microdrive is placed on the chamber after dura mater puncture and the guide tube is lowered using the gross stage into the puncture location, gently depressing, and stabilizing the surrounding tissue (steps 2 and 3). Data transfer cables and grounding wires are connected, and the NP probe is lowered into the cortex using the fine stage (steps 4 and 5). (b) 2D side view lowering schematic to further illustrate probe insertion. (c) Spiking patterns from a cell shifting across the channels during implantation. At time (t = 1 s) spiking activity is detected on a distal contact and as the probe is advanced (t = 1–5 s) the spiking activity shifts to contacts more proximal to the probe base.

Implantation occurs in three general phases: puncturing the dura mater at the desired location using the Narishige microdrive, attaching the Flex MT microdrive and gross lowering of the guide tube system, and lowering the NP probe. Initially, the dura mater was manually punctured using a long, 18-gauge puncture cannula via a puncture assist device in the Narishige (figures 3(a) and (b), Step 1). The Narishige microdrive was then removed to place the Flex MT microdrive on the cephalic chamber.

The Flex MT microdrive positioning ring was kept in a raised position to protect the NP guide tube system while fixing the microdrive to the cephalic chamber. Next, the ball screw was turned to descend the positioning ring and electrode drive tower with attached NP guide tube system (figures 3(a) and (b), Step 2). As the 3 mm guide tube approached the dura mater surface (estimated with measurements from the testing apparatus and under visualization with a surgical microscope), the ball screw was slowly turned to press the guide tube into the punctured dura mater hole until the bottom platform of the guide channel slightly depressed the dura surface (figures 3 (a) and (b), Step 3). We found that appropriate placement of the guide tube within the punctured dura was associated with a filling of the guide tube with cerebrospinal fluid.

After seating the NP guide tube system with the ball screw, the EPS drive wire was placed in the fine lowering stage clamp to drive the NP along the channel in micrometer increments (figures 3 (a) and (b), Step 4). EPS depth measurements and movement speeds were controlled via the Alpha Omega software. NP probe movement speed was approximately 50 *µ* s^−1^ during implantation. The distance traveled in one movement was 0.5 mm while the probe was above the guide tube, 0.25 mm per movement within the guide tube, and 0.1 mm per movement in cortical tissue. The 0.1 mm displacement per movement was to help ensure the probe shank enter the cortical tissue safely. After each movement, a few seconds were taken to assess signal changes across channels and then another movement was initiated. Probe insertion time ranged from 20–30 min in total, including 10 min to lower the probe tip to the opening in the guide tube or cerebral spinal fluid (CSF) and approximately 10–20 min to lower the probe through the guide tube to the final depth, until there was approximately 1 mm distance between the top of the guide tube and probe base. This final depth was chosen because there is a widening in probe width at the point where the probe shank affixes to the probe base and to reduce risk of probe shank breakage. With a guide tube length of 4.5 mm, we estimate an insertion depth of approximately 5 mm past the bottom of the guide tube. A grounding wire was attached to the head post and an interface cable was connected from the recording system prior to probe lowering (figures 3(a) and (b), Step 4) to monitor signals in real time using Open Ephys (Siegle *et al* 2017). The signal noise floor decreased once probe contacts entered the cerebral spinal fluid that filled the guide tube. Neuronal firing was observed once the probe entered brain tissue, and the shift in spiking activity along the recording contacts was monitored as the probe advanced (figure 3(c)), further confirming implantation depth change and probe integrity. The NP probe was advanced to the final depth (figures 3(a) and (b), Step 5); in our recordings, we inserted until ∼1 mm distance remained between the top of the guide tube and base of the probe. Finally, the NP probe holder clamp was fixed in place with a set screw to immobilize the probe (figure 2(a)) for the remainder of the recording.

### 2.6. Data collection and processing

Neural data were collected using NP 1.0 probes or NHP NP 1.0 probes. NHP NP 1.0 probes are identical to the NP 1.0 probes except that NHP 1.0 probes have a shank cross-sectional area of 90 × 100 *µ*m as compared to the 70 × 20 *µ*m shank for the NP 1.0 probe. NP recording technology allows users to select up to 384 of the 960 recording channels to record from at any given time. The 384 channels closest to the tip, which span 3.84 mm, were selected for testing in this study.

Data sampled at 30 kHz and filtered with a 500 Hz high pass filter was spike sorted using Kilosort 2.5 (Steinmetz *et al* 2021) and manually curated using Phy following the guidelines written by S. Lenzi and N. Steinmetz (https://phy.readthedocs.io/en/latest/sorting_user_guide). Briefly, putative single unit (SU) were identified as clusters with waveforms distinct from other waveforms or noise on the same channels and had clear refractory periods. Clusters containing two or more groups of waveforms that were not distinct enough to be reliably separated into individual SU were grouped into a multi-unit (MU) cluster. Clusters with a firing rate lower than 0.1 Hz were excluded from further analysis. Neural data analysis for spiking data and plot generation was performed using MATLAB code modified from the spikes repository made by N. Steinmetz (https://github.com/cortex-lab/spikes).

Behavioral data were collected with two Logitech USB webcams recording at 20 frames per second. One camera recorded eye behavior and the other camera recorded reaching behavior. Cameras were synchronized and video data was saved using a Tucker Davis Technologies (TDT) RZ2 and Synapse software. The NI DAQ chassis (NI PXIe-1071) containing a NP PXIe acquisition card and a DAQ (PXIe-6341, X Series DAQ) were synchronized together with Open Ephys online synchronization and then synchronized with the TDT recording system using a 1 V square wave pulse train at the beginning and end of each recording. Behavioral timepoints during the reaching task were manually extracted in MATLAB (MathWorks, 2021) for use in analysis.

The NHP performed a reaching task where the NHP was trained to hold their hand on a start pad location, reach to one of three positions where food would be presented, bring the food to their mouth, and then return to the starting location. Successful trials are defined as a completion of all steps. Multiple behavioral time points can be extracted from this reaching task, however, for most of the analysis we have focused on examining data with respect to reach initiation. Additionally, the reaching task provided a good test of implant and recording stability due to forces imposed on the system by the primate during the reaching task.

## 3. Results

### 3.1. Summary of experiments

**Figure 4.**
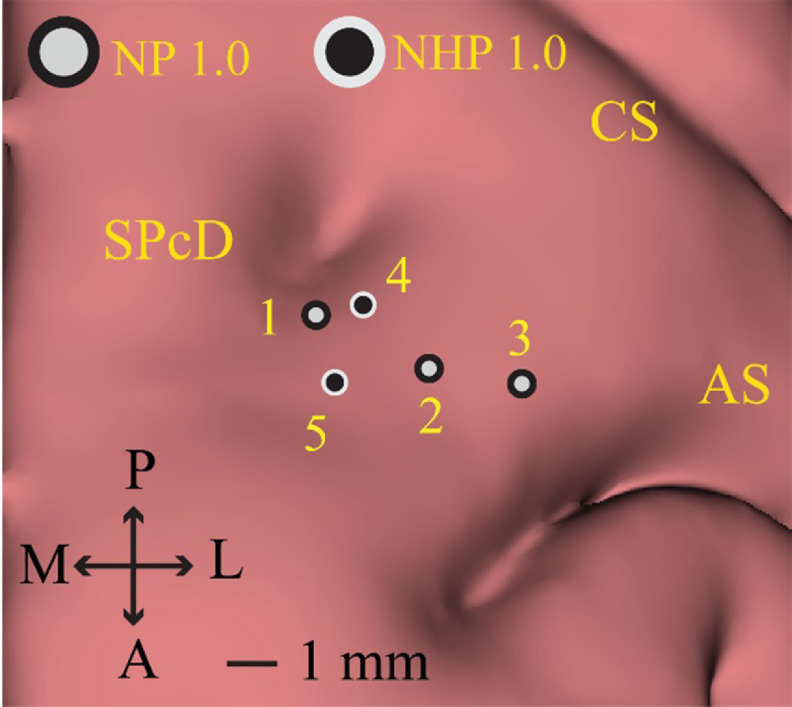
Left hemisphere Neuropixels implant locations NP 1.0 and NHP 1.0 implantations were located within the dorsal premotor cortex (PMd), defined by the region anterior to the central sulcus (CS) and immediately posteromedial to the arcuate sulcus (AS) and anterolateral to the superior precentral dimple (SPcD). NP 1.0 probes implant locations are demarcated by light grey circles with a black border. NHP 1.0 implantation locations are demarcated by black circles with a light grey boarder. The NP 1.0 probe used for recording area 2 was successfully reused for recording area 3; the same NHP NP 1.0 probe used for recording area 4 was reused for recording area 5.

Following the described implant methodology, we performed three implantations using commercially available NP 1.0 probes, two with NHP NP 1.0 probes, and successfully reimplanted both versions of the probe one time (table 1, figure 4). Two NP 1.0 probes and one NP NHP 1.0 probe were used. Experimental sessions were typically 3–4 h in duration, with multiple recording blocks obtained throughout the session. No NP probe broke after insertion into the brain; however, during a second implantation attempt, one probe broke on the dura mater due to a rotation in the X stage while fixing the Flex MT microdrive on the cephalic chamber (i.e. figure 3(a), Step 2). At the base of the probe where the probe shank integrates, there is a widening to ∼0.14 mm. Therefore, we drove the probes to a position where the base of the probe was 1 mm above the top of the guide tube due to concerns of breaking the probe shank on the wider portion. Utilizing Kilosort 2.5 and following guidelines noted in section 2 we identified 1198 (627 SU/571 MU) units across the five recording sessions (table 1).

**Table 1.** Summary of Neuropixels probes implantations and experimental outcomes. For all implantations, neural data was recorded on the 384 channels closest to the tip of the probe at rest and during a reaching task. Offline spike sorting was performed using Kilosort 2.5. Single unit (SU) and multiunit (MU) clusters extracted from the reaching task data across sessions are presented. Implantation locations 1–5 are displayed in figure 4.

Recording area	Probe type	SU/MU clusters during reaching
1. PMd	NP 1.0	124/165
2. PMd	NP 1.0	48/32
3. PMd	NP 1.0	124/111
4. PMd	NHP NP 1.0	202/125
5. PMd	NHP NP 1.0	129/138

### 3.2. Stable NP recordings in a NHP performing a reaching task

**Figure 5.**
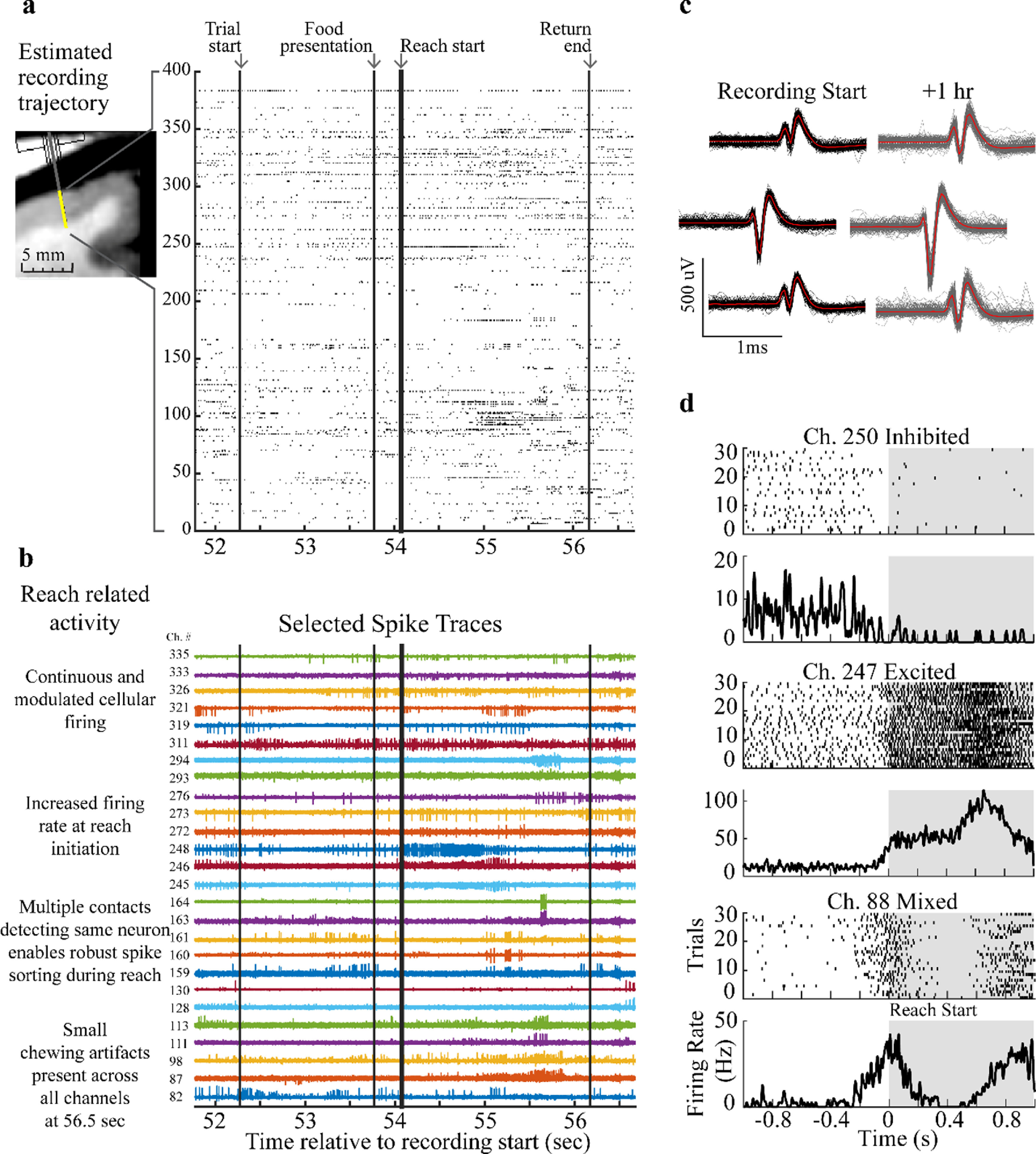
Neuropixels recording in primate PMd cortex during a reaching task. (a) Recording data from 384 recording sites closest to the tip of the probe (yellow) with the recording trajectory and depth approximated with model measurements. A representative example raster plot of SU and MU spike times identified from the Neuropixels probe recording channels during a single reach behavior trial. Trial start, food presentation, reach start and return end are labeled and indicated with vertical lines. (b) Selected spike traces show constant firing, modulated firing, increased firing, bursting, and small chewing artifacts during various aspects of the reach across the array. Signal amplitudes are scaled to the max absolute voltage detected on each channel for the displayed time. (c) Three sample spike waveforms (n = 200, mean in red) recorded from channel 246, 248, and 250 at recording start and at 1 h after recording start. (d) Peristimulus time histograms from three example units aligned to reach start and located at different depths along the probe. All data presented are from recording 5 in the table 1.

To test the ability of our setup to enable stable neuronal recordings, we analyzed data collected while the monkey was actively engaged in a food reach and retrieval task. Neural data were collected from all probes during reaching (table 1). Recording depths for each probe implantation were approximated using the model measurements and physical recording measurements (figure 2). Data were recorded on the bottom (most ventral) 384 channels, displayed as the yellow line in the estimated recording trajectory pane. A raster plot of SU and MU spike times across the length of the probe during a reaching trial are presented in figure 5(a). Behavioral epochs and time points are demarcated by vertical lines and labeled in the raster plot.

Black vertical lines indicate trial start, food presentation, reach start, and the end of the trial (figure 5(a)). Of the 384 channels recorded, a subset of channels was selected to illustrate the types of raw neural traces collected by the probe and the heterogeneous responses captured across the span of the recording channels during the reaching task (figure 5(b)). The same units are held on a group of channels (channels 159–163) throughout the reaching task which enabled robust spike sorting. Small chewing artifacts were present in the signal (at time ∼56.5 s). Bursting activity (channel 163 and 164), tonic firing (channel 311), regions of low or high modulation (channel 82, 87, 98) were present during the reaching task. This showed that the NP probe shank can detect a variety of responses across the column of sampled neural tissue.

Waveforms from an isolated SU were displayed from recordings obtained 1 h apart (figure 5(c)) and show consistency in waveform amplitude and shape throughout the reaching task. Peristimulus time histograms were generated for three SU/MU clusters selected at different depths across the probe and demonstrate three different responses that were observed relative to reach onset: inhibited (SU), excited (MU), and mixed (MU) (figure 5(d)). The sample waveforms (figure 5(b)) and consistent response of cells during each reaching task trial (figure 5(d)) show that, with proper fixation of the recording equipment, this implantation methodology can accomplish stable recordings (e.g. holding cells) throughout NHP reaching behavior.

Supplementary figure 1 shows additional raster plots across multiple behavioral trials. Data from multiple trials further illustrate stability in the method to record using a NP probe. Additionally array raster plots show the variety of the spiking activity at different depths collected using a NP probe in PMd cortex.

### 3.3. Drift trace as a metric of stable recordings

**Figure 6.**
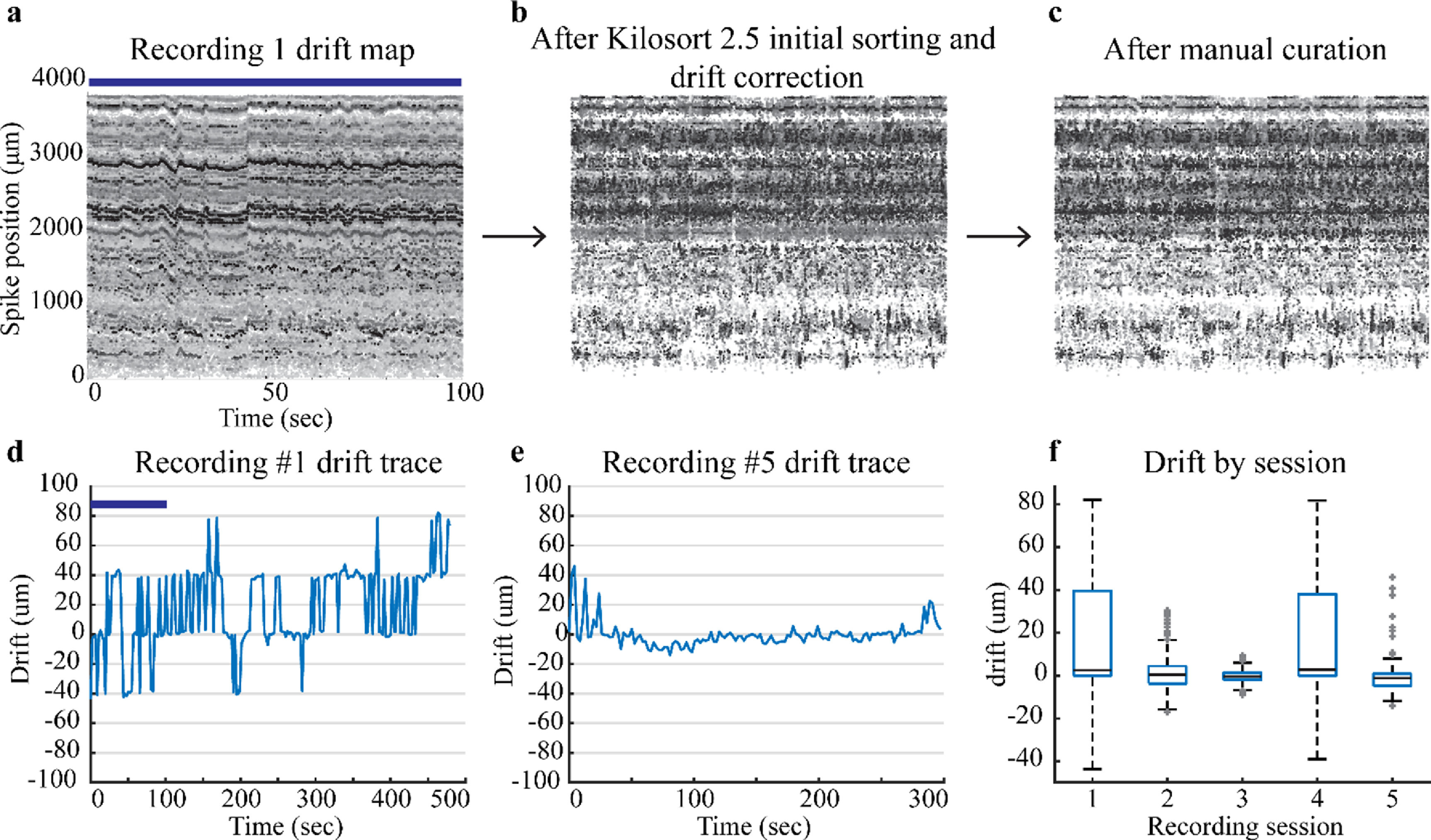
Drift maps and traces for recordings. The top row has drift maps only for Recording 1. (a) Drift maps for Recording 1 display the drift initially assessed by Kilosort 2.5, (b) after Kilosort 2.5 initial sorting and drift correction, and (c) after manual curation with Phy GUI. The blue bar shows the corresponding time in the Recording 1 drift trace and drift map. Darker points have a higher spike amplitude and lighter points have smaller spike amplitudes. (d) Recording 1 drift trace shows a higher amount of drift than (e) Recording 5 drift trace. (f) Box plots display median and quartiles for drift during a reaching task for each recording session listed in table 1.

Two useful outputs of Kilosort 2.5 based on sorted spiking data information include drift maps and drift traces. Drift maps provide a visual assessment of the spike location (figures 6(a)–(c)) and amplitude. Drift traces (figures 6(d) and (e)) are a batch by batch (2.2 ms per batch) calculation of the relative position of the neurons detected on the array. Recording 1 drift map (figure 6(a)) displays the spikes across the 384 channels closest to the tip of the probe as a function of time. Each consecutive drift map (moving left to right) follows a separate step in the spike sorting process and displays the overall drift correction and curation of the spiking data. Figure 6(a), recording 1 drift map, displays data from 0 to 100 s and recording 1 drift trace (figure 6(d)) displays the drift for the entire recording. This data was drift corrected and initially sorted (figure 6(b)) via Kilosort 2.5 and then manually curated following the methods described previously. The total spike count remains similar which is why figures 6(b) and (c) look similar.

Drift data provide the amount of displacement up or down that occurs during a recording session. Kilosort 2.5 (Steinmetz *et al* 2021) best handles slow drift which can be seen as a gradual trend in the drift trace. Slow drift physically occurs when the tissue slowly passes against the NP shank. We allowed the probe to settle for at least 30 min to mitigate aspects of slow drift from the initial probe insertion. Fast drift is a second form of drift which occurs when the probe moves up and down with respect to the tissue. These events are more transient in nature and harder to account for with sorting algorithms (Rey *et al* 2015). Fast drift events, sharp transient peaks in a drift trace, indicate the probe moved with respect to the tissue seen in figure 6(d). These events were better addressed and mitigated after the initial recording session by using a set screw to stabilize the final recording position of the probe. Drift decreased in sessions after the initial recording except for recording 4 (figure 6(f)). Low drift recordings sessions are a marker of stability and expedite the spike sorting process. Drift data from this implantation approach show that this implantation system can accomplish low drift (i.e. stable) recording sessions.

## 4. Discussion

The main aim of this study was to develop and assess a method to implant NP probes in a NHP. In this paper we have demonstrated an innovative approach, the NP guide tube system, which can be used to implant, explant, and reuse NP 1.0 and NHP 1.0 NP probes in NHP’s (rhesus macaque). Not only did this method enable repeat penetrations through thicker dura and provide stable recordings during NHP behavioral reaching tasks but it can also be adapted to most two stage microdrive systems. Given the implantation success rate and adaptability, the method facilitates broader use of the NP recording technology in NHPs.

Previous approaches have used a NaN microdrive with a customized NP holder (Trautmann *et al* 2019) or mixed microdrive components (Wang *et al* 2021). Trautmann *et al* (2019) accomplished the NP implant by visually aligning and inserting the NP probe through an incision in the dura mater. Wang *et al* (2021) developed an approach to implant probes without a guide tube by reinforcing the NP probe shank with a Tungsten wire. Both methods implanted NP probes in animals with newly exposed dura mater. While successful, the first method had the highest risk of breaking the probe on implant and throughout the experimental protocol given that there were no protective measures to ensure probe safety. Additionally, this method also requires visual access to align the probe with the incision. Wang *et al* (2021)’s method reduces the risk of breaking the probe on implantation and during experimental movements by stiffening the NP shank with a tungsten wire; however, the reinforcement process physically damages channels near the probe base and increases the risk of breaking during the wire adhering process. The method described in this paper offers minimized risk of damaging or breaking the probe during implant. The guide tube system enabled repeatable implants in a protective, controlled manner while minimizing implant risks.

NP preparations are most optimally performed after a recent craniotomy, where the dura is at its thinnest (Trautmann *et al* 2019) but dura mater thickens and tissue growth permeates above the dura as a natural side effect after a craniotomy. A strength of the NP guide tube system presented here was that this acute method can be used when dura is thicker. At the end of this study histological measurements confirmed the dura and tissue growth within the recording chamber ranged from 1.5–3 mm thick. By altering the length of the guide tube on the guide channel, this system could be customized to bridge dura mater of varying thickness. NHP 1.0 versions are commercially available with larger cross-sectional areas to make the probe less fragile; however, increasing the cross-sectional area of the probe does not mean probes can be implanted without a guide tube or durotomy. These larger NP probes have been utilized in two recent intraoperative studies in humans (Chung *et al* 2022, Paulk *et al* 2022), with relatively large craniotomy exposures and probe insertion after a durotomy was made. The NP guide tube system can implant both the NHP 1.0 probes and NP 1.0 probes. Given the guide tube system’s compatibility with both probes, implanting NP 1.0 probes may be preferable to reduce tissue damage during probe insertion. We did not observe any salient differences in recording performance between probe types, though a larger number of recordings would be warranted to confirm this observation. Finally, the NP guide tube system sets up a base methodology to implant longer NHP probes. Simply swapping the gross lowering tower to a taller tower, printing a longer guide channel, and assembling the system would provide a system that can implant longer probe versions. A four shank version of the NP probe, NP 2.0 (Steinmetz *et al* 2021), could also be adapted to this NP guide tube system with two modifications. The probe holder would require a redesign to hold the NP 2.0 probe and a larger diameter guide tube would be required. For example, the guide tube could be machined from a 15-gauge needle with an inner diameter of 1372 *µ*m (302 *µ*m wider than the NP 2.0).

Using NP probes, we can identify more cells and assess cell responses (localized to a specific contact) at different depths and with longer probes across multiple structures simultaneously. NP technology will be useful for assessing layer specific neuronal processing in various NHP cortical areas. These data presented here provide insight into the capabilities offered by implanting a NP probe in a NHP and illustrate several of the features implanting high density probes in primates.

## 5. Limitations and future directions

The main success of this study was the ability to implement a guide tube system to perform acute NP recordings in an awake behaving NHP. There are several limitations, however. The first limitation is the use of a separate dura puncture device. Incorporating the puncture equipment into the same system used to insert the probe would provide a reduction in components, remove alignment steps, and save time during preparation. This is an engineering challenge that should be addressed in future iterations of this implantation methodology. Similarly, condensing the two-step lowering procedure into a single, finely controlled lowering system would be beneficial as it would reduce the number of required steps and improve implant depth resolution by reducing the stacked error in measurements. Condensing both steps into a single lowering stage is, however, challenging from an engineering and design perspective. Despite having two separate systems, the current methodology is straightforward to implement. The goal for next iterations is to further simplify the procedure by removing alignment steps and streamlining the lowering procedure.

We processed a limited amount of data from each probe to assess probe stability and to evaluate some simple measures of brain activity, however, the NP probe offers great opportunities to implement new analysis that leverage high numbers of simultaneously recorded cells, e.g. to examine neural population dynamics during reaching (Churchland *et al* 2012, Trautmann *et al* 2019). Additionally, combination of analytical approaches such as current source density may be beneficial as a future direction for physiological determination of cortical lamina and investigation into layer-specific neuronal processing (Bastos *et al* 2018).

## 6. Conclusion

NP probe technology has permeated into various animal models because of the ability to record hundreds of neurons simultaneously across different depths. Implantation methodology and recordings in NHPs is still relatively new for this probe technology. By addressing implantation concerns with the NP guide tube system, this high-density recording technology can be fully utilized. While the NP probes require careful planning to prevent breaking on implant, the system presented here can accomplish stable electrophysiology recordings for two versions of the NP probes. Furthermore, this system can reimplant probes, thus improving the usability of the NP probes in NHPs. The NP guide tube system has provided a successful implant method for NP probes in NHPs and will provide a robust implant method for other researchers interested in NHP NP probe recordings.

## Data Availability

The data cannot be made publicly available upon publication because the cost of preparing, depositing and hosting the data would be prohibitive within the terms of this research project. The data that support the findings of this study are available upon reasonable request from the authors.
